# Editorial: Recent advancements in mycobacterial diseases research

**DOI:** 10.3389/fmicb.2024.1504449

**Published:** 2024-10-16

**Authors:** Matt D. Johansen, Jotam G. Pasipanodya, Selvakumar Subbian, Vishwanath Venketaraman

**Affiliations:** ^1^Centre for Inflammation, Faculty of Science, School of Life Sciences, Centenary Institute and University of Technology Sydney, Sydney, NSW, Australia; ^2^Department of Medicine, Vanderbilt University Medical Center, Nashville, TN, United States; ^3^Public Health Research Institute, New Jersey Medical School, Rutgers University, Newark, NJ, United States; ^4^Department of Basic Medical Sciences, Western University of Health Sciences, Pomona, CA, United States

**Keywords:** mycobacterial diseases, TB, NTM diseases, *Mycobacterium tuberculosis*, non-tuberculous mycobacteria, drug resistance, diagnosis, therapies

## Introduction

According to the World Health Organization, the number of global tuberculosis (TB) cases in 2021 reached 10.6 million, a 4.5% rise from the number of cases recorded in 2020 (*World Health Organization Global Tuberculosis Report-2022*). This indicates the urgent need to develop measures to curtail the disease. The diverse host immune response to *Mycobacterium tuberculosis* (Mtb), the causative agent of TB, is influenced by both host and bacterial factors and the immune response of the host to infection (Berrington and Hawn, 2007).

A pathologic hallmark of Mtb infection in humans is the formation of granulomas, a highly organized cellular structure at the site of infection (Kumar et al., 2024; Klever et al., 2023). Although granulomas are considered host-protective because they restrict Mtb into a confined space, the bacteria can utilize those granulomas to escape from killing by host immune cells. Thus, the fate of Mtb and the course of infection and disease progression is largely determined at the level of granulomas (Subbian et al., 2015; Warsinske et al., 2017; Gideon et al., 2022). Granulomas are heterogeneous, comprised of a variable mixture of immune and non-immune cells as shown in the spectrum of human TB disease and recapitulated in various animal models of Mtb infection. However, granulomas consistently evolve, undergoing caseous necrosis and cavitation in active pulmonary TB patients, however, this is only observed in non-human primates and rabbit models of Mtb infection (Kumar et al., 2024; Klever et al., 2023). While cavitary granulomas facilitate bacterial dissemination, highly cellular “solid” granulomas contain bacteria at the core. Importantly, the granulomas are encased by a layer of fibrotic material, comprised of various types of collagens and elastin secreted by the macrophages and fibroblasts surrounding the granulomas (Subbian et al., 2015; Warsinske et al., 2017; Gideon et al., 2022). These fibrotic cores leave residual parenchymal tissue damage, which hinders successful antibiotic penetration into the granuloma to kill Mtb. However, the mechanism of collagen formation and the nature of collagen fibers that constitute the fibrotic zone of granulomas are not completely understood. Here, Song et al. describes an automated quantitative assay to determine fibrosis characteristics of various granuloma types using 16 tissue types of TB patients and lung sections from a Marmoset model of Mtb infection. The authors have used a conventional Masson trichrome staining method to visualize the extent of fibrosis in different types of lung granulomas. The authors used a novel, stain-free second harmonic generation (SHG) two-photon excited fluorescence (TPEF) microscopy to further characterize the nature of fibrosis in the granulomas. Results from these analyses show that in the fibrotic area, aggregated collagens, made of short and thick clusters of 200–620 nm in size were the predominant form, compared to the long and thick disseminated collagens of 200–300 nm size. Furthermore, MMP-9 which codes for matrix metalloproteinase-9, was found to be upregulated in the granulomas of various tissues. This study contributes a valuable quantitative imaging tool to gain insight into the fibrotic dynamics of TB granulomas. Further exploration and refinement of such tools to understand the mechanism of fibrosis in TB granulomas would facilitate the development of novel strategies to prevent TB sequelae and efficiently kill Mtb within those granulomas.

The host immune cell recognizes mycobacteria through various pattern recognition receptors (PRRs), such as the Toll-like receptor (TLR). TLR recognizes pathogen-associated molecular patterns (PAMPs), including lipoprotein and lipopolysaccharides that are present in bacteria (Jahantigh et al., 2013). TLR1 recognizes tri-acylated lipopeptide, and TLR6 recognizes di-acyl lipopeptide (Buwitt-Beckmann et al., 2005). TLR1 recognizes the 19 kD lipoprotein of Mtb (Takeuchi et al., 2002), and TLR2 recognizes 19 kD lipoproteins, lipoarabinomannan and other Mtb PAMPs (Brightbill et al., 1999). In this Research Topic, a study by Varshney et al. showed an association between *TLR2* deletion *(*−*196 to* −*174)* and *TLR1 743 A* > *G* gene polymorphism and drug-resistant pulmonary TB in a population from Agra, Uttar Pradesh, India. The findings from this study suggested that in the present population, the heterozygous (*Ins/Del*) genotype and deletion allele of *TLR2 deletion (*−*196 to* −*174)* polymorphism are associated with increased risk for the development of drug-resistant TB. Furthermore, for *TLR1 743 A* > *G* gene polymorphism, A/G genotype, and G allele are found associated with healthy controls, suggesting a protective role against TB which warrants further investigation.

The Research Topic also includes a study by Choreño-Parra et al. that aims to investigate the utility of the Systemic Immune-Inflammation Index (SII), Fibrinogen, and T-SPOT.TB in distinguishing between active pulmonary tuberculosis (PTB) and non-tuberculous lung diseases. This study concluded that SII and Fibrinogen are positively correlated with the degree of tuberculosis inflammation and the bacterial load. The combined detection of SII, Fibrinogen, and T-SPOT.TB is significant in distinguishing between active PTB with positive T-SPOT.TB results and non-tuberculous lung disease as described by Yu et al.. The proteolytic activity of A Disintegrin and Metalloproteinase 17 (ADAM17) regulates the release of tumor necrosis factor (TNF) and TNF receptors (TNFRs) from cell surfaces. These molecules play important roles in TB shaping innate immune reactions and granuloma formation. This Research Topic also includes study findings suggesting a role for SNPs of ADAM17 in genetic susceptibility to TB.

In addition to TB, non-tuberculous mycobacteria (NTM), also called “atypical mycobacteria” or “mycobacteria other than TB (MOTT) pose significant challenges to human health, particularly among vulnerable populations worldwide (Gopalaswamy et al., 2020). These ubiquitously present organisms were once considered environmental and opportunistic bacteria, however, recent clinical studies indicate that diseases due to NTMs are rising globally. Due to its overlapping characteristics with Mtb, such as acid-fastness and mechanism of pathogenesis, the diagnosis and transmission of NTM are underestimated in endemic populations (Gopalaswamy et al., 2021). Similarly, there are limited effective treatments currently available for NTM infections, although several drugs are in the process of being repurposed. However, most first-line anti-TB drugs are ineffective in clearing NTM infections. Thus, despite the continued emergence of NTM infections, currently available measures to diagnose or treat NTM infections are underdeveloped and require urgent attention. There are over 200 NTM species described to date whose prevalence varies significantly, depending on global geographic regions and populations. Importantly, only a small proportion of NTM species are opportunistic pathogens, mainly *Mycobacterium avium* complex (MAC), *Mycobacterium abscessus* complex (MABC), and *Mycobacterium kansasii* (*M. kansasii*), which collectively are known to account for the majority of human NTM infections. Furthermore, MAC, MABC, and *M. kansasii* account for >95% of NTM pulmonary disease (NTM-PD). In up to half of pulmonary NTM cases, the infecting bacteria do not cause invasive disease, rather, they just colonize the tissue. This ability to colonize host tissues without causing invasive disease ultimately complicates both the diagnosis and treatment of NTM infections. Furthermore, the clinical significance of NTM colonization, and in particular the risk factors predisposing individuals to NTM infection are poorly described and are still an enigma to healthcare providers in the clinic and researchers in laboratories. Indeed, even when NTM do cause pulmonary diseases, in ~10% of such cases more than one NTM species is recovered from sputum samples, which is usually either MAC or MABC (Wallace Jr et al., 1998). Additionally, both organisms have been shown to form biofilms in lung lesions and to persist within diverse microbial communities, particularly in patients with pre-existing lung diseases such as bronchiectasis, previous TB, or chronic obstructive pulmonary disease (Yamazaki et al., 2006). These are significant knowledge gaps that pose challenges for NTM pharmacology and microbiology.

In the review article by Conyers and Saunders, the current challenges and prospects for the treatment of NTM were elaborately discussed. Various strategies adapted to determine the current treatment regimen and the challenges involved in such approaches to treat infections caused by different species of NTM were explained with relevant literature support from the late 1990s to the early 2000s. Importantly, this review summarizes five clinical trials conducted to evaluate the efficacy of inhaled liposomal amikacin with other adjunct antibiotics for the treatment of NTM infections and concludes that additional studies on drug optimization are needed to address potential adverse effects due to drug-drug interactions in the combination therapy. Furthermore, this study analyzed 15 active clinical trials focused on the optimization of treatment regimens for NTM infection and highlights challenges such as antibiotic resistance, drug-susceptibility testing, and bacterial physiologic measures, including biofilm formation, colony morphology, and complications associated with bacterial cell wall for effective drug therapy. Finally, the review discusses novel therapeutic modalities such as phage therapy and newly approved antibacterials to combat NTM infections. These measures should help to effectively control NTM diseases worldwide.

A study by Meliefste et al. provided an extensive review of the literature surrounding *M. abscessus* biofilm characterization. In their review, they highlighted the significant differences in biofilm culture methods and techniques that exist and have been applied to *M. abscessus* biofilm studies. Furthermore, the authors also discuss the importance of *M. abscessus* strain selection and the role this plays in biofilm formation, with previous studies concluding that only the smooth morphotype forms biofilms, however subsequent studies have since demonstrated that the rough morphotype can indeed form biofilm and that these disparities between studies are largely due to differences in experimental conditions and strains. The authors also discussed the role of culture medium (Middlebrook 7H9, Synthetic Cystic Fibrosis Medium) and specific constituents such as Tween 80, Iron, and Magnesium. Finally, the authors also discussed drug activity testing on *M. abscessus* biofilms, with indications that traditional anti-mycobacterial drugs are relatively ineffective against *M. abscessus* biofilms, highlighting the significant global challenge in the treatment of chronic *M. abscessus* lung infections.

Another study by Narimisa et al. involved a systematic review and meta-analysis of the prevalence of *M. kansasii* in clinical and environmental isolates. Among 6,640 publications identified, only 134 (118 for clinical and 16 for environmental) met the criteria for inclusion for the analysis of *M. kansasii* prevalence. For the analysis of *M. kansasii* in environmental samples, two studies examining prevalence in soil found a prevalence rate of 0.5%, while 15 studies examined prevalence in water samples and found *M. kansasii* prevalence to be 6.4%. Overall, *M. kansasii* prevalence in environmental samples was found to be 5.8%. When examining geographical prevalence, *M. kansasii* prevalence was found to be highest in Europe at 12.1% and lowest in North America at 2.6%, while prevalence was shown to have significantly increased from 4.9% from 1990–2000 up to 8.9% in 2021–2022. Overall, this study highlighted that the prevalence of *M. kansasii* is significantly higher than originally thought and demonstrates the need for increased surveillance and effective management and infection control strategies.

Similarly, Nguyen et al. have provided a comprehensive review article summarizing antibiotic resistance mechanisms of *M. abscessus*. The authors emphasize the underpopulation of the *M. abscessus* drug pipeline, largely due to current drug discovery approaches that rely on anti-mycobacterial drug library screening which are largely ineffective due to intrinsic resistance mechanisms of *M. abscessus*. Further, the authors highlighted both the extensive intrinsic resistance traits of *M. abscessus*, as well as the novel acquired resistance traits occurring due to spontaneous mutations occurring at specific genes due to the presence of antibiotics following extensive exposure. In this review, the authors discuss antibiotic modifying/inactivating enzymes such as aminoglycosides, β-lactams, rifampicin, and active derivatives (such as rifabutin), as well as target-modifying enzymes such as macrolides, and the extensive repertoire of efflux pumps that *M. abscessus* possesses to extrude antimicrobials of different classes. Finally, the authors discuss acquired resistance traits of *M. abscessus* against newly discovered compounds such as MmpL3 inhibitors and emphasize the urgent need to expedite the discovery of new antimicrobial classes for the treatment of *M. abscessus* to overcome intrinsic and acquired resistance mechanisms.

Tan et al. performed comparative genomic and microbiological analyses to further our understanding of siderophore iron acquisition mechanisms in both environmental and respiratory isolates of NTMs from Hawai'i. Iron is a critical element required for many physiologic processes; hence it is tightly regulated by both the human host and mycobacterial pathogens. Therefore, the hypotheses put forward by Tan et al. are pertinent in explaining NTM diversity across geography and related virulence patterns, especially in the context of drug and immune tolerance, and the ability of the pathogen to persist within granulomatous lesions. In this study, the authors identified *M. abscessus, M. porcinum*, and *M. intracellulare* subsp. *chimaera* (hereafter referred to as *M. chimaera*) with a total of 51 isolates (28 respiratory and 23 environmental) used for further analysis. Overall, the authors found that respiratory NTM isolates showed a significantly higher mean number of mycobactin (*mbt*) compared to non-pathogenic environmental species. The exception was with *M. chimaera* from Hawai'i, which naturally had a higher number of *mbt* genes because of the higher iron content in the environment. Furthermore, ESX-3 is a Type VII secretion system possessed by mycobacteria with a known role in iron acquisition, and in this study, the authors showed that the number of *ESX-3* genes was significantly increased in environmental *M. chimaera* isolates as compared to respiratory isolates but remained unchanged for both *M. abscessus* and *M. porcinum*. Finally, the authors performed *in vitro* siderophore assays under low iron conditions and showed that both *M. abscessus* and *M. porcinum* grew better than *M. chimaera* which was in contradiction to the KEGG analysis. The authors concluded that further studies are required to understand the role of iron acquisition in NTM isolates and the contribution of the host respiratory conditions in driving iron siderophore utilization.

In summary, articles in this Research Topic have raised more questions than those answered by the data presented and have identified significant knowledge gaps that need to be addressed within the mycobacterial field. Specifically, and as pointed out by the authors, the role of iron acquisition in NTM clinical isolates raised questions as to whether dietary iron supplements would ameliorate or exacerbate infections. Further, are these observations restricted to NTM that grow on agar as well as those that only grow in liquid media? Would the expression of iron siderophore genes present an advantage to clinical isolates *in vivo*? In addition, correlating these physiological findings to clinical markers of NTM virulence such as bacterial growth rate, colony morphology, and phenotype, or drug minimum inhibitory concentrations, would be critical for the application of these findings to combating NTM infections globally. The Research Topic of intrinsic resistance and drug tolerance to currently used chemotherapeutic agents is the focus of three reviews in the series. These are the “known unknowns” upending the recommendations for standardized treatments by the American Thoracic Society (ATS)/Infectious Disease Society of America (IDSA) and European Society for Clinical Microbiology and Infectious Disease (ESCMID). Although MABC is the focus for two of these reviews, the themes and lessons apply to MAC as well. Macrolides such as clarithromycin and azithromycin, and aminoglycosides like as amikacin form the backbone of chemotherapy for NTM infection treatment and management. Until recently, there had been no substantive pharmacokinetics (PK) and pharmacodynamics (PD) studies performed to inform drug doses and dosing schedules for these organisms. Most currently used therapy regimens and doses were developed from those used for Mtb, despite heterogeneous clinical phenotypes and the fact that many NTM species are highly drug-resistant. For that reason, optimal drug combinations are unknown, therapy durations are poorly defined, and therefore, clinical outcomes are universally poor (Pasipanodya et al., 2017a,b). The current clinical trial efforts targeting repurposed anti-tuberculosis drugs described by Conyers and Saunders in this series are notable developments. But they are stopgap efforts. The lasting solution is multiple regimens targeting each NTM organism. As mentioned earlier, each of these NTM species is distinct with variable minimum inhibitory concentrations and causes distinct clinical diseases (pulmonary vs. cutaneous vs. disseminated NTM disease). Therefore, PK/PD-informed and designed regimens with targeted drug doses that acknowledge the PK variability of the population and PD variability of mycobacteria represent the gold standard and should be adopted to rapidly accelerate drug discovery and the identification of next-generation antimicrobial therapies.
